# Examining the Effectiveness of a Case Management Program for Custodial Grandparent Families

**DOI:** 10.1155/2012/124230

**Published:** 2012-06-07

**Authors:** Lenora Campbell, Dana L. Carthron, Margaret Shandor Miles, LaShanda Brown

**Affiliations:** ^1^Division of Nursing, Winston-Salem State University, Winston-Salem, NC 27402-6172, USA; ^2^School of Nursing, The University of North Carolina at Chapel Hill, Chapel Hill, NC 27599, USA

## Abstract

Researchers have identified complex needs of custodial grandparent families and lack of access to needed resources such as housing, financial and legal assistance, and health care. Case management links these families with needed services while helping them develop skills to promote their health and well-being. This paper describes a case management program for custodial grandparent families using a nurse-social worker case management team. data were collected from 50 grandparents and 33 children using surveys and semi-structured instruments. Physical and mental health outcomes were measured using Short Form-12 Health Survey (SF 12) to measure the perceived quality of health for grandparents and the Child Behavior Checklist to measure the emotional and behavioral functioning of grandchildren. Grandparents more positively perceived their mental health after participating in the program. Perceptions about physical health were generally the same before and after the program. Grandparents' reported that many grandchildren had emotional and behavioral problems in the clinical range. These findings highlight the need for further research on the mental health needs of children being parented by grandparents as well as determining effective models and interventions to minimize adverse effects of parenting on grandparents.

## 1. Introduction

Over the last two decades, the number of children raised by relatives in what is known as “kinship care” has grown exponentially, and most of the caregivers are grandparents. For many of these grandparents, raising a grandchild can be a long commitment [1]. While being custodian to grandchildren has positive aspects (e.g., the potential to guide the next generation into healthy and productive lives), research suggests that it can also be very stressful. With little opportunity to plan, grandparents are thrust unexpectedly into this challenging new role and confronted with a myriad of problems and issues in parenting their grandchildren. The challenges come out of complex social situations, including the death or unstable functioning of their adult children and the health and behavioral problems of the grandchildren [2].

 Grandparents who are rearing grandchildren are often elderly and with low income and thus come into the custodial role with their own challenges. Many have chronic health problems, and the stress of caring for grandchildren increases their risk for cardiovascular and other health problems [3, 4]. Caregiving leaves little time or money to meet their own health and emotional needs [2, 5]. Also, grandchildren in custodial grandparent homes are an at-risk population. Many have been exposed to difficult life circumstances such as the death of parents or parents who are drug addicted or incarcerated. Some have experienced violence, abuse, and neglect [6, 7]. Thus, these children are susceptible to physical, behavioral, and mental health problems that compromise their growth and development.

 Few studies have examined the health and well-being of children in custodial grandparent families, but findings from these studies consistently point out that they are at risk for emotional, behavioral, cognitive, and social problems [2, 8]. Thus, while grandparents may constitute a good safety net that diverts children from the child welfare and foster care system, parenting these children can be especially challenging. There is clearly a need for interventions to help grandparents manage their parenting role, while enhancing their health and well-being, as well as the health and well-being of their grandchildren. However, few interventions have been developed to assist grandparents in their caregiving role. 

 Case management, a process that links individual and family needs to resources, has been identified as a possible model for helping these grandparents [9]. Case management involves a collaboration between participants in the process of assessing, planning, implementing, coordinating, monitoring, and evaluating the options and services required to meet an individual's needs. Case managers function to mobilize, coordinate, and maintain services and resources [9] and to empower clients and families for self-care. For many custodial grandparents and their families, case management can be an important service linking these families with needed services, housing, financial and legal assistance, health care, and other resources [10]. This paper describes a case management intervention with custodial grandparents and examines the outcomes, including grandparent and child well-being.

## 2. The Grandparenting Program

The Grandparenting Program in Winston-Salem, North Carolina, implemented the System of Care (SOC) case management model, a family-centered, strength- and empowerment-based program designed to enhance the well-being and successful functioning of custodial grandparents and their grandchildren, based on Project Healthy Grandparents [9]. Families were recruited for the program from the surrounding county. Grandparents had to be the primary caregiver for their grandchildren, and grandchildren had to be under 18 years of age. Families enrolled in the Grandparenting Case Management (GCM) Program for one year. The goal of the case management program with custodial grandparents was to empower them to develop their own abilities and to find and use resources that would improve their health and well-being and the health and well-being of their grandchildren. 

 Families (grandparents and their grandchildren) were followed by a nurse and a social worker who constituted the case management team. The case management program included (a) assessment of the strengths and needs of the family, (b) goal setting to develop the case management plan, (c) implementation, and (d) evaluation of the plan and outcomes. This paper presents a description of the program and reports a study designed to evaluate its effectiveness.

### 2.1. Assessment

 Nurses and social workers completed separate assessments to identify the characteristics of families and individuals, their strengths, resources, and needs. Nurses assessed the health history and health status of grandparents and grandchildren using standardized and investigator-developed measures as well as physiological assessments (e.g., height/weight, BMI, blood pressure, and glucose and cholesterol levels). The health behaviors and health perceptions of grandparents were also assessed, and a developmental assessment was completed on children 6 years of age and under. Social workers assessed family composition, characteristics, and history. In addition, social workers assessed the mental and emotional health of grandparents and grandchildren, using standardized measures. Both nurses and social workers assessed the client's access to services and resources to meet individual and family needs. 

 Grandparents were helped to identify personal and community strengths and resources and to understand that every person, family, and community has strengths, and while their personal situations might be challenging, they may also reveal important strengths and opportunities. In this way grandparents began to develop a greater awareness of the personal, family, and environmental supports available to them as they dealt with challenging situations. 

 An important goal of the assessment phase was the development of a trusting and professional relationship with the family. It was important that families perceived case managers as trustworthy, nonjudgmental, and helpful since many of these grandparents had had distrustful relationships with health, school, and social service agencies and, initially, were reluctant to accept case managers.

### 2.2. Goal Setting and Development of Case Management Plan

In order to effectively link families with needed services and resources and assist them to improve their health and well-being, case managers worked with the family to develop a comprehensive family plan with goals and objectives, specific interventions, and desired outcomes. The goal setting process began with identification of strengths by the family and case managers, who helped the grandparents see how their lives reflected a number of strengths. The next step was identification of individual and family goals. Grandparents were encouraged to build upon their strengths as they identified activities to meet goals. 

 The family plan was then presented to a multidisciplinary team made up of the case managers, grandparents, and individuals and agencies reflected in the family plan. Team members were asked about the role their agency could play in helping grandparents achieve their goals or to comment on the aspects of the plan in their area of expertise or service. Once approved, the family plan served as the basis for case management services to the family. As the needs of the families and individual family members changed, case managers and families made changes to the plan.

### 2.3. Implementation

 Most of the parents were substance abusers prior to the birth of the child. The age range of caregivers was 35 to 77 years, with a mean age of 56. One-third had not completed high school. Slightly more than a third worked either part or full time. Almost 60% had an income less than $15,000, despite the fact that the average household had approximately two grandchildren; most received no additional resources for the care of their grandchildren. Although all kinship caregivers can receive Temporary Assistance for Needy Families (TANF), less than half received this assistance. Slightly more than a fourth received child support. Almost one-half (46%) received food stamps. Many of these resources come through the child welfare agency but families were reluctant to involve child welfare in their efforts to obtain resources because of concern about the possible consequences, for example, the grandchild being taken from the home and placed in foster care. 

 Over the course of one year, nurses and social workers provided at least monthly visits to grandparents (or monthly phone calls, depending on the need) to meet the goals and objectives of the family plan. The monthly case managers' visits were sufficient for approximately half of the families. A number of families, however, required at least bimonthly visits, and approximately 25% required weekly visits for an extended period of time. The intensity of the intervention was based on the needs of the families and the resources available to them. Seven of the 68 families were maintained in the program longer than the prescribed year. The decision to continue services beyond the year was based on the continued needs of the family and made by mutual consent of the grandparent and the case management team. All families received health-related services, skill building around self-care, parenting, effective advocacy and effective communication, and problem-solving and assistance in accessing needed resources. Case managers provide referrals to families for services needed to achieve goals and followed up with caregivers to determine whether referral appointments were kept and whether the referral was effective. When referrals were not kept, barriers were assessed and grandparents were assisted with problem-solving to reduce the barriers. 

 Case managers also provided learning aids to enhance caregivers' understanding of age-appropriate behaviors of grandchildren and indicators of normal growth and development. They helped the grandparents work through issues with their adult children, including determining whether or not (or how best) to integrate adult children back into their parenting role. 

 The goal of case managers was to provide the grandparent with initial assistance until he or she was able to master the necessary skills to sustain the effort. To facilitate this, case managers helped grandparents anchor their progress; that is, they helped the clients recount what they had gone through, what they had done to make progress, and how differently it felt now. This helped grandparents process the experience and understand how they were changing and what was being done to help them make changes. 

 Grandparents were also provided a weekly small group skill-building program. Weekly evening meetings focused on (a) enhancing parenting skills; (b) advocating and accessing services; (c) improving health and wellness; (d) parenting children with special needs. Weekly groups for children focused on academic assistance and personal and social enrichment activities.

Case managers' greatest efforts were spent on connecting families with needed services. This required a significant amount of time to cultivate relationships with service providers and help grandparents locate needed services such as housing, legal aid, health care, and emergency assistance for daily sustenance. Case managers found it essential to have strong relationships with various service providers, child welfare, school system, counseling or mental health agencies, health care providers, and other community-based agencies. Representatives of a number of these agencies served on our advisory committee and were profitable for connecting families with needed services.

### 2.4. Evaluation/Outcomes

Case Managers evaluated the goals of each family and its individual members and reported on progress in monthly notes and in the discharge summary. Progress on goals was also reported during bimonthly case management meetings, which were used to help case managers identify and resolve problems, ensure that issues were not overlooked, and identify additional opportunities to support families. Case managers also completed postprogram surveys so that the effectiveness of the program could be determined.

## 3. Evaluation of Program Effectiveness

### 3.1. Methods

#### 3.1.1. Sample

 Over the 3 years the program was in operation, 68 families enrolled. The grandparents were primarily African Americans who were single, divorced, or widowed. Two of the families were headed by grandfathers and two by great-aunts (hereafter referred to as “grandparents”). Grandparents were the primary caregivers for 1 to 10 children (with an average of 2.3). Most of the children were girls (54%). Primary caregiving status was based on whether grandparents and grandchildren resided in the same home and whether grandparents provided the majority of caregiving. Potential participants were informed of the case management program through word of mouth and unpaid radio, newspaper, and television advertisement. Institutional IRB approval was obtained prior to enrolling participants in the program. Potential participants telephoned the project office where they were provided with information about the program, and those who agreed to participate were assigned a case management team. During the first home visit, participants signed an informed consent.

### 3.2. Data Collection

 To determine the effectiveness of the case management program, we collected pre- and postdata on grandparents' quality of life using the Short Form-12 Health Survey (SF-12) and grandparents' perceptions of the behavioral and emotional functioning of their grandchildren using the Child Behavior Checklist.

 The SF-12 assesses two aspects of quality of life—physical and mental health. Using a Likert-type rating scale, respondents are asked about their ability to perform certain tasks (e.g., walking up a flight of stairs) or the degree to which physical or mental factors limit activities. The scale has eight subscales measuring physical functioning, physical role, bodily pain, general health, vitality, social functioning, and role/emotional and mental health. Two summary scores can also be computed: a physical component score and a mental component score. The 8-scale scores and 2 summary scores are transformed from the rating scale to a 0 to 100 standardized score. Higher scores indicate better health-related quality of life. The SF-12 has demonstrated reliability and validity in older adults and ethnic groups [11–13].

 The Child Behavior Checklist (CBCL) for 6- to 18-year olds was used to measure the behavioral, social, and emotional functioning of the children. Grandparents rated their grandchildren on how true each item was now or within the past 6 months, using the following scale: 0: *not true as far as you know*; 1: *somewhat or sometimes true*; 2: *very true or often true. * Grandparents also provided information on 20 competence items covering the child's activities, social relations, and school performance [14].

 The Competence Profile includes three scales: Activities, Social and School, as well as a Total Score. Activity scores reflect the child's involvement in activities such as sports or other recreational activities, jobs, and chores, and the quality and amount of the child's participation. The Social scale includes participation in organizations, number of close friends, number of weekly contacts with friends, how well the child gets along with others, and how well the child works and plays alone. The School scale assesses the child's performance in academic subjects, involvement in remedial services, failures, and other school problems. The Total Competence score is the sum of the three scale scores [14]. 

 The Syndrome Profile includes 8 syndromes: Anxious/Depressed, Withdrawn/Depressed, Somatic Complaints, Social Problems, Thought Problems, Attention Problems, Rule-Breaking Behavior, and Aggressive Behavior. The CBCL also groups syndromes into two broad groups: Internalizing and Externalizing. Internalizing problems include the syndromes Anxious/Depressed, Withdrawn/Depressed, and Somatic Complaints, problems that are mainly intrapersonal. Externalizing problems include syndromes that highlight interpersonal conflicts such as aggressive behavior and rule breaking [14]. The scales are based on factor analyses of parents' ratings of 4,994 clinically referred children and were normed on 1,753 children aged 6 to 18. 

 Data were collected by nurses and social workers trained in the data collection process. Data from the project instruments were entered into an SPSS data base by a trained member of the program staff. Descriptive statistical tests were used to describe the demographic and health characteristics of grandparents and grandchildren, as well as the behavioral and emotional functioning of children. Paired *t*-tests were used to compare pre- and posttest scores for health-related quality of life in grandparents and emotional, behavioral, social, and cognitive functioning of grandchildren.

#### 3.2.1. Results

 The 49 families for whom we had complete outcome data included 50 caregivers, nearly all (96%) of who were grandmothers, African American (83%), and unmarried (74%). They ranged in age from 42 to 77 (*M* = 56.7, SD = 8.8), and 78% were high school graduates. The majority were unemployed (77%), and some received disability benefits (31%). Almost a fourth (23%) did not have health insurance. The most prevalent health problems of grandparents were vision impairments (58%), arthritis (54%), chronic pain (41%), and hypertension (30%). The grandparents were taking care of an average of 1.5 children (SD = .83), for a total of 73 children. Almost half (46%) had an annual income of less than $15,000 and received no supplemental assistance. Only 22 (42%) were receiving TANF/AFDC, and less than a fifth (19.2%) were receiving child support. Grandchildren had been living with grandparents for 3 to 181 months. Only one grandparent reported having legal guardianship of the grandchild.

 Only the grandchildren who were school age were included in this study (*n* = 33) since outcome data for younger children were not collected. The primary reason grandparents were taking care of grandchildren included parental abandonment (32.5%), parent drug abuse (27.5%), and incarceration (17.5%). More than half of the grandparents reported that the children had been abused or neglected by at least one parent (53%). Almost 30% reported that the grandchildren had been prenatally exposed to illicit substances, but many of the grandparents were not able to respond definitively to this question. The children's ages ranged from 6 to 15 (*M*  = 9.4; SD = 4.3) and they were evenly divided between girls and boys. The great majority of grandparents (92.3%) reported that the grandchildren had health insurance. Grandparents reported both chronic and acute health problems among the children. The most common were asthma (22%), vision problems (20%), headaches (18%), attention deficit disorder (15%), enuresis (15%) and rashes (15%).

#### 3.2.2. Quality of Life of Grandparents

 Over half of the custodial grandparents rated their health as good to excellent (62%) with the rest rating their health as fair to poor. Their mean scores on the physical health and mental health subscales before and after participating in the case-management program are presented in Table 1. Perceptions of physical health did not significantly differ from pretest to posttest (*P* ≥ .05). Indeed, for role functioning and vitality, grandparents' health perceptions slightly decreased, indicating that, in those areas, grandparents actually rated their health worse after case management than before. However, grandparents' perceptions of their mental health significantly improved from pretest (*M* = 53.25) to posttest (*M* = 63.25; *P* ≤ .001).

#### 3.2.3. Social, Emotional, and Behavioral Problems of Grandchildren

 Based on the report of grandparents, almost half (*n* = 15) of the grandchildren scored in the clinical range on the Total Competence scale of the CBCL (Table 2). Children were more likely to receive scores in the clinical range on the Activities and Social scales, suggesting that they were less likely to be involved in sports, recreational, or job-related activities and had poorer quality involvement in social activities than children of the same age in the normative sample. Children in the sample were also more likely to be reported to have academic problems than a national sample of their peers.

 For behavioral and emotional problems, nearly a fourth of the children in the sample had scores in the clinical range on the externalizing scale (*n* = 8); 4 children scored in the clinical range for rule breaking and four for aggressive behaviors. Although the numbers were small, grandparents reported fewer symptoms at the end of the case management program than at the beginning. Fewer children were identified as scoring in the clinical range on internalizing behaviors, though there was little change from pretest to posttest.

## 4. Discussion and Implications for Practice

 Custodial grandparents and the children they parent constitute an at-risk population but effective intervention can minimize their at-risk status. However, while a number of studies have identified the physical and emotional health problems experienced by grandparents, little research has been conducted on interventions to mitigate these adverse effects. This study evaluated the use of case management as a potential strategy. 

 The case management intervention targeted the health and well-being of grandparents. While their quality of life, as measured by the SF 12, improved over the course of the case management program, the changes were statistically significant only for the mental health score. A number of factors might explain the lack of significance for the physical scores. First, the majority of caregivers rated their health as excellent at the initial assessment (62%), despite the fact that most had multiple chronic health problems. Such a positive initial assessment provided little opportunity for improvement. Second, an important objective of the case management program was to identify health problems and to assist caregivers to seek appropriate care. Focusing caregivers on their health might have led them to adopt a more realistic assessment of their health. Further, a third of the caregivers in the program did not have health insurance. As a result, health problems may not have been fully addressed because of lack of access to appropriate health care. This is consistent with previous studies that have shown a lack of resources available to custodial grandparents, especially those who are not in the child welfare system [5]. Mental health perceptions of the caregivers may have been more directly impacted by the case management intervention and the weekly groups may both have provided needed support. 

 According to the grandparents in this study, a number of children had behaviors that, at the very least, warranted further assessment. Further, almost 40% of the grandparents reported behaviors suggesting that children experienced significant competency problems, primarily in the areas of activities and school. Neither of these findings is surprising, and both are consistent with the literature, which has noted both economic and academic disadvantages for many children in custodial grandparent families. This helps to explain the slight decrease in the activities score and the lack of improvement in the school score after participation in the case management program. A fifth of the grandparents reported behaviors suggesting significant problems such as anger, violence, defiance, and rule breaking. Grandparents reported fewer of these behaviors, however, at the completion of the program. Grandparents were less likely to identify internalizing behaviors of grandchildren (e.g., withdrawn and depressed); other studies have found similar findings among other populations [15]. Clearly, externalizing behaviors are more disruptive to the child's environment and, consequently, are more likely to be recognized. Case managers observed improved relations between grandparents and grandchildren and fewer complaints about grandchildren's behavior at the end of the program.

 The case management program did not directly work with children to reduce their social, emotional, or behavioral problems, and thus it is not surprising that some of the scale scores did not improve after case management. Although few studies have compared the well-being of children in kinship care to the general population of children [16], some studies have shown that children in kinship care families, primarily custodial grandparent families, have some of the same characteristics, problems, and needs as children in nonkin foster care families [2]. Further research is needed to better understand the needs of this population of children and to develop more targeted interventions to improve their health and well-being. Any additional research should include a comparison group to control for the effects of time and maturation.

 Studies have found that while custodial grandparents provide stability for children there are adverse health effects of parenting on custodial grandparents [3, 4]. Clearly more research is needed to identify effective interventions to maintain the benefits of custodial grandparent families while minimizing the adverse impacts of parenting on their health and well-being.

## 5. Conclusion

While several studies have attempted to identify interventions to enhance the health of grandparents, no study has been found to determine effective interventions for the children in their care. Clearly, healthcare providers need to be aware of the unique circumstances of these children and the ways these circumstances might impact their well-being. Health care providers also need to be aware of the impact of custodial caregiving on grandparents and should incorporate interventions, including education strategies, in their health care. Evaluation of the GCM Program suggests that this might be an effective model for providing custodial grandparent families with needed resources and support. The use of the case management teams comprised of nurses and social workers is effective in improving grandparents' perceptions of their mental health functioning in improving relations with their grandchildren. 

Research-based guidelines are needed to provide direction for how best to provide health care to this population. Further research is needed to help frame policies that provide for the needed resources for this group, including health care.

## Figures and Tables

**Table 1:** Grandparents' perceived QOL before and after case management (*N* = 50).

Subscales	Pretest	Posttest
	Mean (SD)	Mean (SD)
Physical	55.5 (35.8)	53.5 (38.1)
Role	57.8 (32.4)	59.8 (28.6)
Bodily pain	59.0 (33.8)	61.0 (37.5)
General health	49.0 (24.6)	49.8 (26.2)
Vitality	48.5 (28.3)	46.5 (28.6)
Social	72.0 (28.4)	71.5 (29.9)
Role/emotional	62.3 (31.4)	73.8 (28.2)
Mental health	53.3 (13.1)	63.3 (19.6)
PCS	55.3 (24.0)	55.3 (26.3)
MCS	59.8 (19.1)	63.8 (20.3)*

*P < .001.

**Table 2:** Children 6–18 scoring in clinical range on CBCL (*N* = 33).

Subscales	Before case		After case	
	management		management	
	*N*	%	*N*	%
Total competence	9	27.3	7	21.2
Externalization	6	18.2	2	6.1
Activities	5	15.2	4	12.1
Total problems	5	15.2	1	3.0
Thought problems	4	12.1	0	—
Internalization	3	9.1	2	6.1
Aggression	3	9.1	1	3.0
Withdrawal	3	9.1	0	—
Attention	2	6.1	0	—
Anxious	2	6.1	0	—
Social problems	2	6.1	3	9.1
School	2	6.1	1	3.0
Somatic	1	3.0	0	—
Rule breaking	1	3.0	0	—
